# Effects of MEK-inhibitor treatment in infants with lymphatic abnormalities in noonan syndrome

**DOI:** 10.3389/fped.2026.1842723

**Published:** 2026-07-15

**Authors:** J. Wagenpfeil, K. Hoß, A. Henkel, D. L. Kütting, J. A. Luetkens, A. Mueller, C. C. Pieper, A. Groteklaes

**Affiliations:** 1 Department of Diagnostic and Interventional Radiology, Division for Minimally-invasive Lymph Vessel Therapy, University of Bonn, University Hospital of Bonn, Bonn, Germany; 2 Center for Rare Congenital Lymphatic Malformations, Center for Rare Diseases, University of Bonn, University Hospital of Bonn, Bonn, Germany; 3 Department of Neonatology and Pediatric Intensive Care, University of Bonn, University Hospital of Bonn, Children’s Hospital, Bonn, Germany

**Keywords:** central conducting lymphatic anomaly, lymphedema, MEK-inhibition therapy, MR lymphangiography, noonan syndrome, trametinib

## Abstract

Noonan syndrome (NS) is a genetic disorder associated with dysregulation of the RAS/MAPK signaling pathway and is frequently accompanied by lymphatic abnormalities. These lymphatic complications can lead to severe clinical manifestations, including chylous effusions and respiratory compromise, particularly in infants. Management of such complications is often challenging, and conventional therapies may be insufficient in severe cases. Recently, targeted therapies affecting the RAS/MAPK pathway, such as MEK inhibitors, have emerged as a potential treatment option for patients with refractory lymphatic disease. In this context, the present study describes our initial clinical experience with MEK inhibitor therapy using Trametinib in infants with Noonan syndrome and lymphatic abnormalities. Six infants with genetically confirmed NS (3 female, 3 male; mean age 0.5 ± 0.72 years) presenting with lymphatic abnormalities and intractable chylous effusions were treated with Trametinib. Clinical presentation was assessed at baseline as well as at 6-week and 1-year follow-up. Dedicated MR lymphangiography was performed at baseline and after one year, and lymphatic abnormalities were rated using a 6-point Likert scale. Possible adverse events were documented. After one year of therapy, considerable clinical improvement was observed in all patients. None of the patients required chest drainage and no clinical signs of chylothorax were present. Follow-up MR lymphangiography demonstrated a decrease in pleural effusions in all cases, with no residual fluid in three patients and only minimal residual fluid in the remaining three. Pathologic pulmonary and pleural lymphatic perfusion decreased in all cases (median 3 vs. 1). However, lymphatic imaging did not demonstrate normalization of lymphatic anatomy or flow in any patient. Overall, in this exploratory analysis Trametinib therapy resulted in considerable clinical improvement in infants with lymphatic abnormalities associated with Noonan syndrome, while MR lymphangiography showed only limited remodeling of the central lymphatic system over one year of treatment without normalization of lymphatic anatomy or lymphatic flow.

## Introduction

Noonan syndrome (NS) is a congenital autosomal dominant, multisystem disorder with short stature, facial dysmorphisms, skeletal abnormalities, congenital heart disease, and lymphatic anomalies (1–6). Lymphatic manifestations include peripheral lymphedema, protein-losing enteropathy (PLE), pulmonary and intestinal lymphangiectasia, chylous ascites, and chylothorax (6).

Pathogenic variants along the RAS–MAPK pathway —most commonly *PTPN11*, *RIT1*, *SOS1* and *RAF1*— lead to overactivation of *ERK1/2*, implicated in lymphatic development (2, 7–9). Excessive *ERK* activation therefore is thought to cause lymphatic abnormalities in NS (1, 6, 9). Dedicated lymphatic imaging has shown extensive central abdomino-thoracic abnormalities even in patients with localized signs, including thoracic duct (TD) dysplasia, impaired central run-off, and pathological chylo-lymphatic reflux consistent with central conducting lymphatic anomaly (CCLA) (5). These are readily evaluated by dynamic-contrast enhanced MR-lymphangiography (DCMRL).

There is no accepted treatment strategy for the lymphatic phenotype of NS, and refractory chylous effusions often persist despite medical, interventional, or surgical therapy (6). Trametinib, a MEK1/2-inhibitor developed for malignancies driven by RAS-MAPK activation, is a promising option for NS with symptomatic lymphatic abnormalities (10, 11). Single cases in NS with hypertrophic cardiomyopathy or lymphatic disease showed encouraging results (1, 11–18), but systematic data on Trametinib and central lymphatic changes beyond case reports are lacking.

The aim of this study was therefore to report experiences of treating infants with NS and lymphatic abnormalities with refractory chylous effusions with Trametinib.

## Material and methods

### Patient cohort

Children were included into this retrospective study when they had

- Genetically confirmed NS,
- A history of lymphatic abnormalities (lymphedema/chylothorax/chylous ascites/pulmonary or intestinal lymphangiectasia/lymphorrhea),
- Current refractory chylo-lymphatic effusions unresponsive to conservative treatment,
- MEK-inhibitor therapy with Trametinib for at least one year,
- Nodal DCMRL at our institution, before and after one year of therapy and
- Clinical follow-up data during treatment.

The indication for off-label Trametinib therapy was reached in a dedicated interdisciplinary board for the treatment of rare lymphatic diseases. DCMRL was performed as part of the in-house standard clinical work-up in patients with suspected lymphatic disorders. Parents or legal guardians were informed in detail about the clinical procedures, including the off-label use of contrast-agent and drugs employed in treatment and provided written informed consent for the clinical treatment. The institutional review board approved retrospective data analysis of existing clinical and imaging data and waived the requirement for additional informed consent for the retrospective research use of these data.

Between 05/2020 and 01/2022 six pediatric patients with NS (3 female, 3 males; mean age 0.5 ± 0.72 years [range 2 month—2 years]) and lymphatic abnormalities were included into the study. Causative gene variants were pathogenic variants of *PTPN11* (*n* = 2), *RIT1* (*n* = 3) and *KRAS* (*n* = 1). All patients presented with refractory chylothorax, 2/6 with chylous ascites and 1/6 with chylopericardium. 2/6 patients additionally had lower extremity peripheral lymphedema, which was assessed clinically. See Table 1 for patient characteristics.

**Table 1:** Clinical presentation at baseline.

Patients	1	2	3	4	5	6
Clinical baseline characteristics at birth						
Gender	F	F	M	M	F	M
Gestational age at birth (weeks)	34	37	37	41	29	27
Birth weight	3,050	4,090	3,000	3,658	2,150	1,200
Fetal nuchal edema	No	No	No	No	No	Yes
Fetal pleural effusion	Yes	Yes	Yes	Yes	Yes	No
Fetal ascites	No	No	No	No	No	No
Hydrops fetalis	Yes	No	No	No	Yes	No
Polyhydramnion	No	Yes	No	No	No	No
Molecular diagnosis						
Affected gene	*RIT1*	*RIT1*	*KRAS*	*PTPN11*	*PTPN11*	*RIT1*
Protein-level amino acid change	p(Ala77Thr)	p.Phe82Leu	p.Pro34Arg	p.Asp106Ala	p.Asn308Ser	Phe99Leu
Variant in cDNA sequence	c.229G > A	c.246T > G	c.101C > G	c.317A > C	c.923A > G	c.297T > G
Initial clinical presentation						
Cardiac abnormalities	PS	HCM	HCM	HCM	PS	
					VSD	PS
					ASD	HCM
					HCM	ASD
Lymphatic abnormalities	PeLE	CT	CT	CT	PeLE	CT
				CP	CT	
	CT			CA	CA	
Other clinical features						
Facial features	Yes	Yes	Yes	Yes	Yes	Yes
Feeding dificulties	Yes	No	Yes	Yes	Yes	Yes
Hydrocele testis	No	No	Yes	No	No	No
Nondescensus testis	No	No	No	Yes	No	Yes

PS, pulmonary stenosis; HCM, hypertrophic cardiomyopathy; VSD, ventricular septal defect; ASD, atrial septal defect; PeLE, peripheral lymphedema; CT, chylothorax; CP, chylopericardium; CA, chylous ascites.

Initially, all patients underwent conservative treatment for chylo-lymphatic effusions (19) including placement of drainage tubes to relieve effusions, fluid substitution and MCT-diet/parenteral nutrition for a minimum of two weeks. Propranolol was initiated in all patients at 2 mg/kg/day in 2–3 divided doses, and 4/6 patients additionally received Sirolimus (0,8 mg/m^2^/day). When no clinical improvement was observed Propranolol and/or Sirolimus therapy was discontinued before Trametinib was started. The initial oral Trametinib dose was 0.02 mg/kg/day with individual adjustments of the dose depending on the occurrence of side effects (11, 20) (Table 2).

**Table 2:** Treatment and clinical follow up.

Patients	1	2	3	4	5	6
**Treatment**						
Trametinib	Yes	Yes	Yes	Yes	Yes	Yes
Age at start Trametinib treatment	0 y 6 m	0 y 3 m	0 y 4 m	0 y 3 m	0 y 6 m	2 y
Dose of Trametinib (mg/kg/day)	0.02 mg/kg/d	0.02 mg/kg/d	0.02 mg/kg/d	0.02 mg/kg/d	0.02 mg/kg/d	0.02 mg/kg/d
Treatment adherence	Yes	Yes	Yes	Yes	Yes	Yes
Deviations from treatment plan	Dose reduction (0.01 mg/kg/d)	No	No	No	No	No
Duration of Trametinib treatment	1 y 1 m	1 y	1 y	1 y	1 y 1m	1 y
**Previous medication**						
Propranolol	Yes	Yes	Yes	Yes	Yes	Yes
Sirolimus	Yes	Yes	No	No	Yes	No
Sildenafil	No	No	Yes	No	No	No

4/6 patients were previously included in a study focusing on clinical and imaging findings in patients with lymphatic abnormalities in NS before therapy (5). There therefore is some overlap regarding baseline clinical, genetic and imaging characteristics (i.e., imaging findings on T2w and DCMRL at first presentation before treatment) of these patients in both studies. However, the current study focusses on treatment effects and changes in clinical and imaging findings.

### Patient monitoring

Patients were initially monitored until pleural effusions resolved and they could be discharged. Thereafter patients were regularly monitored clinically after 6 weeks of Trametinib treatment and then every three months in our outpatient clinic for congenital lymphatic malformations. Clinical findings and adverse events were documented at the respective time points. To monitor possible morphological and/or functional changes of the central lymphatic system, DCMRL was performed before and one year after treatment. Peri-interventional complications were recorded if present.

### Imaging protocol

DCMRL was performed on a 1.5-T system (Ingenia; Philips Healthcare, Best, The Netherlands) (12) under deep sedation. The patient was placed in supine position on a detachable MR-table into a dedicated pediatric coil. Non-contrast heavily T2-weighted (T2w) imaging was acquired employing a 3D free-breathing high-spatial-resolution, isotropic turbo spin-echo-sequence (TR 3,000 ms, TE 600 ms, flip angle 90°, field of view 400 mm, acquired voxel size 1.2 × 1.2 × 1.2 mm, reconstructed voxel size 0.6 × 0.6 × 0.6 mm, acquisition time 4:45 min). Thereafter the patient was transferred to an ultrasound unit for sonography-guided placement of 22-gauge needles (BD Medical, USA) into inguinal lymph nodes as described previously (12, 21–23) using a linear 18 MHz probe (LOGIQ Vivid E90, GE Healthcare). All lymph node punctures were performed by the same interventional radiologist (C.C.P. with 13 years' experience). After that, the patient was transferred back into the MR-scanner and contrast-enhanced MRL was performed with continuous and slow application (0.5 ml per minute) of 1.0 mmol/mL gadobutrol (Gadovist, Bayer Healthcare, Germany) diluted with physiological saline (1:1). For dynamic imaging, a coronal T1-weighted multi-echo gradient-echo-sequence was repetitively acquired (TR 5.2 ms, TE 1.8 ms and 4 ms, flip angle 20°, field of view: 430 mm, matrix: 480 × 480 mm, acquisition time 40sec) during contrast-injection.

### Data analysis and definitions

Medical records were retrospectively reviewed to gather relevant baseline history including gene-variant, associated congenital heart disease and clinical history of lymphatic abnormalities. Effusions were considered chylous/lymphatic, if lymphocytes were more than 80% and/or triglyceride levels were >110 mg/dl on laboratory examination (23).

Possible side effects of Trametinib including immune suppression, skin, cardiac or hepatic toxicity, ophthalmologic complications, coagulopathy, pancreatitis as well as rhabdomyolysis were evaluated clinically regarding occurrence and severity.

Using the radiological picture archiving system, MR-lymphangiograms were reviewed by two radiologists in consensus (C.C.P and J.W. with 13 and 7 years' experience, respectively) blinded to patients' clinical data. T2w-imaging was evaluated regarding the presence and extent of ascites, pleural or pericardial effusions, edema of the thoracic wall as well as pulmonary lymphangiectasia. DCMRL was evaluated regarding the presence of a TD, chylo-lymphatic leakages as well as pathological chylo-lymphatic reflux. As described before (5), chylo-lymphatic reflux was defined as reversal of lymph flow away from central lymphatics/the TD and the venous termination.

Clinical status and imaging results were compared between baseline and one-year-follow-up after Trametinib treatment. For comparison, the extent of edema, chylo-lymphatic reflux as well as lymphatic perfusion were rated in a 6-point-Likert-scale (0: none to 5: extensive) before and after treatment.

### Statistical analysis

Statistical analysis was done using SPSS, version 27.0 (IBM, Armonk, NY, USA) and GraphPad Prism, version 8.0.2 (GraphPad Software Inc, Boston, MA, USA). Given the small sample size, all analyses were considered descriptive and exploratory. Descriptive statistics were used to summarize patient characteristics, clinical follow-up data and imaging results. Continuous variables are reported as mean and standard deviation, whereas discrete variables are reported as counts, medians and ranges, as appropriate. Changes in imaging parameters during treatment were additionally assessed in an exploratory manner using the Wilcoxon signed rank test. Resulting *p*-values were interpreted cautiously as descriptive indicators of within-patient directional consistency rather than confirmatory evidence of treatment effects. No adjustment for multiple comparisons was performed, and the findings should therefore be regarded as hypothesis-generating.

## Results

### Clinical outcome

Clinical follow-up is summarized in Table 3. After six weeks of Trametinib treatment, pleural effusions reduced considerably in all children so that pleural drainage catheters were removed in 5/6 cases at 6-week follow-up. Chylous ascites present in 2/6 patients also decreased in both patients. On ultrasound the patient with chylopericardium only showed a minimal pericardial effusion. Noticeable peripheral lymphedema in 2/6 patients was unchanged at 6-week-FU. Side effects of Trametinib were seen in 3/6 patients suffering from atopic dermatitis which was combined with peanut/soya allergy in 1/6 and with keratosis pilaris in 1/6. One patient additionally developed slightly elevated liver enzymes. Due to these encouraging treatment results, Trametinib was continued in all infants (with all patients still receiving treatment at the time of data analysis). In 1/6, the dose was reduced to 0.01 mg/kg/d because of the atopic dermatitis; the remaining 5/6 patients continued to receive 0.02 mg/kg/d.

**Table 3:** Clinical follow up.

Patients	1		2		3		4		5		6	
Lymphatic abnormalitis	6 week FU	1 year FU	6 week FU	1 year FU	6 week FU	1 year FU	6 week FU	1 year FU	6 week FU	1 year FU	6 week FU	1 year FU
**Clinical presentation**												
Lymphedema	Unchanged	Unchanged	No	No	No	No	No	No	Unchanged	Unchanged	No	No
PLE	No	No	No	No	No	No	No	No	No	No	No	No
Chylothorax	Reduction	Minimal	Reduction	Minimal	Reduction	No	Reduction	Minimal	Reduction	Minimal	Reduction	No
Chylopericardium	No	No	No	No	No	No	Minimal	No	No	No	No	No
Chylous ascites	No	No	No	No	No	No	Reduction	No	Reduction	No	No	No
**Adverse events/side effects**												
Atopic dermatitis	Yes	Yes	No	No	Yes	Yes	Yes	Yes	No	No	No	No
Peanut and soya allergy	Yes	Yes	No	No	No	No	No	No	No	No	No	No
Keratosis pilaris	No	No	No	No	Yes	Yes	No	No	No	No	No	No
Elevated liver enzymes	No	No	No	No	Yes	No	No	No	No	No	No	No

After one year of Trametinib therapy, pleural effusions had resolved completely in 2/6 patients and almost completely in 4/6 cases not necessitating a drainage-catheter. Chylous ascites and chylopericardium previously present in 2/6 and 1/6 cases, respectively, resolved completely. Peripheral lymphedema again was clinically unchanged compared to baseline. All patients were cared for on an outpatient-basis and took part in life as normal.

Atopic dermatitis remained constant in 2/6 patients and improved slightly after dose reduction in 1/6. In 1/6 patients elevated liver enzymes normalized. 3/6 infants had no side effects during the whole time period. No other adverse events were observed.

### MRL findings

Given the small sample size, all analyses were considered descriptive and exploratory. Detailed imaging findings are given in Table 4. Prior to therapy, T2-weighted MRI and DCMRL were successfully performed in all six infants. Chylothorax and pulmonary lymphangiectasia were present in all cases (median severity rating for both: 3, range 2–4). Chylous ascites present in 2/6 patients was rated as 2 and 3, respectively. Chylopericardium observed in 1/6 cases was minimal at baseline. All cases presented with thoracic wall edema (median 2, range 2–3). DCMRL showed that the TD was completely absent in 1/6 patients, while it was partially absent in the remaining 5/6 cases. Pathological chylo-lymphatic reflux was present in all cases and was most pronounced into intercostal, mediastinal and peribronchial lymphatics (all with a median rating of 3, range 2–3) and led to lymphatic perfusion of pleura and lungs in all cases. Reflux into cervical lymphatics was observed in 3/6 patients with a median severity of 1 (range 0–3). There was no active chylo-lymphatic leakage.

**Table 4:** MRL findings, (-) none, (+) minimal, (++) moderate, (+++) pronounced, (++++) extensive.

Patients	1		2		3		4		5		6	
Age at time of lymphangiography	0 y 2 m	1 y 7 m	0 y 3 m	1 y 2 m	0 y 2 m	1 y 3 m	0 y 2 m	1 y 3 m	0 y 6 m	1 y 7 m	2 y	3 y
**MRL Findings**	**Pre—Treatment**	**1 year FU**	**Pre—Treatment**	**1 year FU**	**Pre—Treatment**	**1 year FU**	**Pre—Treatment**	**1 year FU**	**Pre—Treatment**	**1 year FU**	**Pre—Treatment**	**1 year FU**
**T2-weighted imaging**												
Thoracic wall edema	++	+	++	+	++	+	++	+	+++	++	+++	+
Chylothorax	+++	+	+++	+	++	-	++	+	++++	++	+++	−
Pulmonary lymphangiectasia	+++	+	+++	++	+++	+	++++	++	++	+	++	+
Chylopericardium	−	−	−	−	−	−	+	−	−	−	−	−
Chylous ascites	−	−	−	−	−	−	+++	−	++	−	−	−
**DC-MRL**												
**Thoracic duct**	Absent		Partially absent		Partially absent		Partially absent		Partially absent		Partially absent	
**Leakage**	−	−	−	−	−	−	−	−	−	−	−	−
**Chylo-lymphatic reflux**												
Peripheral	++	+	−	−	−	−	−	−	−	−	−	−
Abdominal wall	−	−	−	−	++	+	−	−	++	+	−	−
Mesentery	−	−	−	−	+++	++	−	−	−	−	+++	+
Mediastinum	+++	+	++	+	+++	++	++	+	+++	+	+++	++
Peribronchial	+++	+	+++	+	+++	++	++	+	++++	++	+++	++
Intercostal/pleural	+++	+	+++	+	+++	++	++	+	+++	++	+++	+
Internal mammary	+++	+	−	−	++	+	−	−	+++	+	−	−
Superficial thoracic	++	+	−	−	−	−	−	−	+++	+	−	−
Cervical	+++	+	+++	+	++	+	++	+	++++	++	++++	++
Axilla	−	−	−	−	++	+	−	−	+++	+	−	−

**Figure 1:**
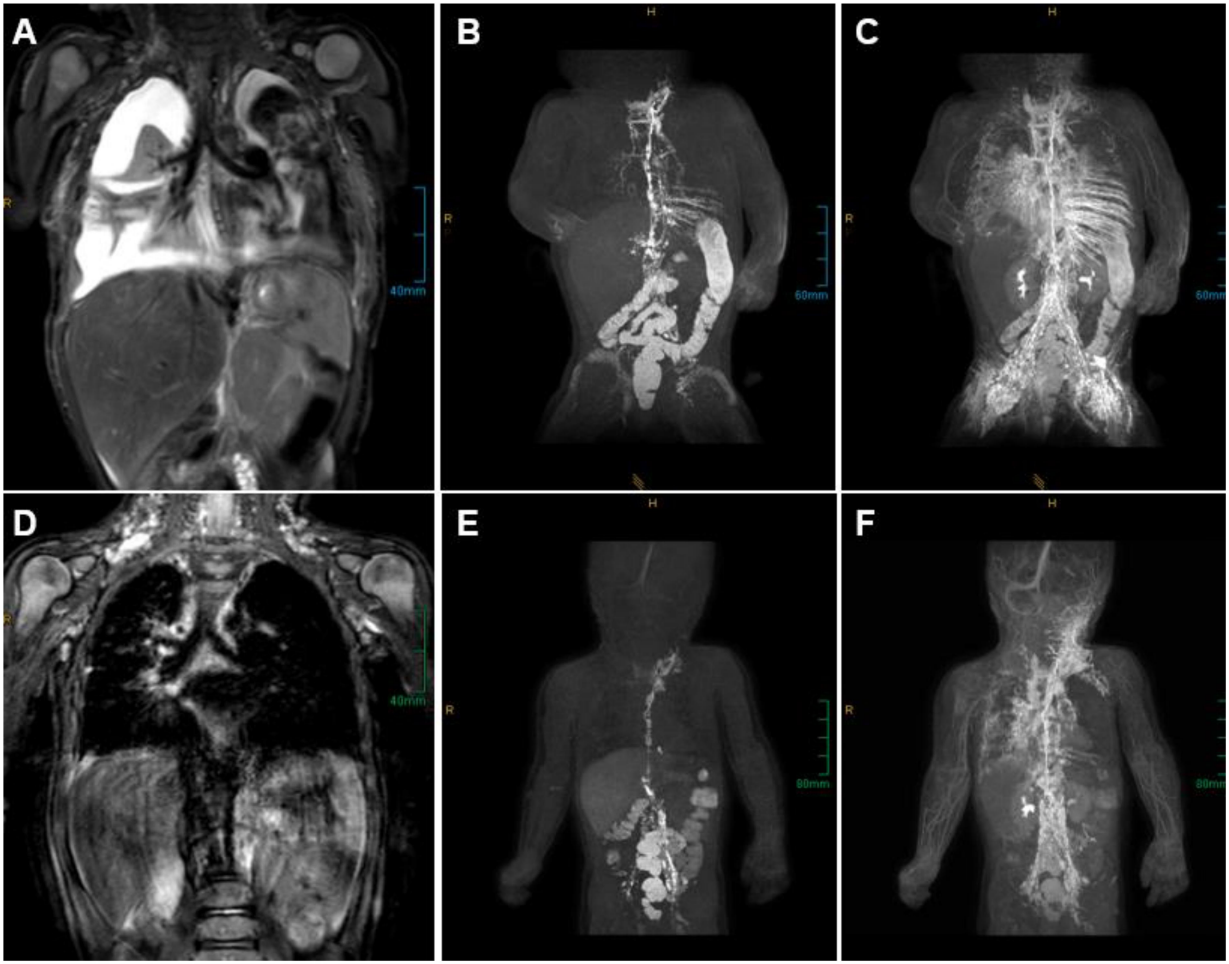
MR-lymphangiography of a 6-month old girl with noonan syndrome (*PTPN11* pathogenic variant) and refractory chylothorax. Coronal T2-weighted mDixon image (water image) demonstrates pleural effusions **(A)** Coronal maximum-intensity projections (MIP) of a contrast-enhanced MR-lymphangiogram demonstrate thoracic duct dysplasia (with aplasia of the terminal part of the duct) and initial reflux into the left pleura and into peribronchial lymphatics in the early phase **(B)** and massive reflux into pleural, pulmonary and cervical lymphatics in the late phase **(C)** After one year of treatment with Trametinib T2-weighted imaging shows improvement of pleural effusions with minimal residual effusions (not visible here) **(D)** While the early phase of contrast enhancement **(E)** demonstrates clear improvement of central lymphatic flow with considerably less reflux in comparison to pre-treatment imaging, the late phase **(F)** shows reduced, but still clear and marked remaining reflux into pleural, pulmonary and cervical lymphatics.

**Figure 2:**
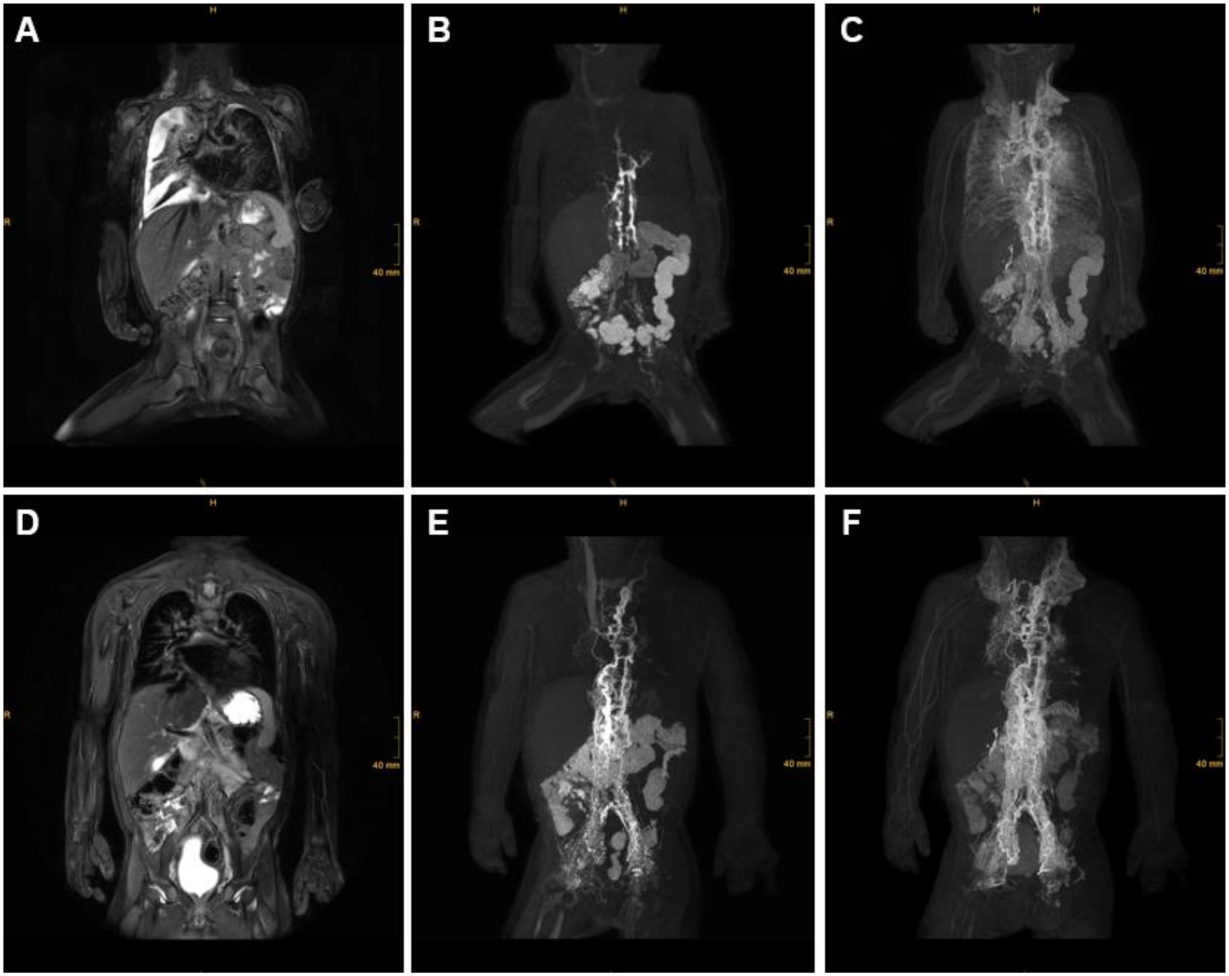
MR lymphangiography of a 2-year-old boy with noonan syndrome (*RIT1*-pathogenic variant) and refractory chylothorax. Coronal T2-weighted mDixon image (water image) shows a large pleural effusion on the right **(A)** Coronal maximum intensity projections (MIP) of a contrast-enhanced MR-lymphangiogram in the early phase shows tortuous lymphatic vessels without a continuous thoracic duct **(B)** This results in massive reflux into the pleural, pulmonary, and cervical lymph vessels in the late phase **(C)** After one year of treatment with Trametinib, T2-weighted imaging shows resolution of chylothorax **(D)** The early phase of contrast enhancement **(E)** continues to show evidence of the markedly tortuous and progressively dilated vessel in the position of the thoracic duct. The late phase shows substantially reduced, but still visible chylo-lymphatic reflux into mediastinal and cervical lymph vessels **(F)** Especially reflux into intercostal/pleural lymphatics is considerably reduced after one year of treatment.

**Figure 3:**
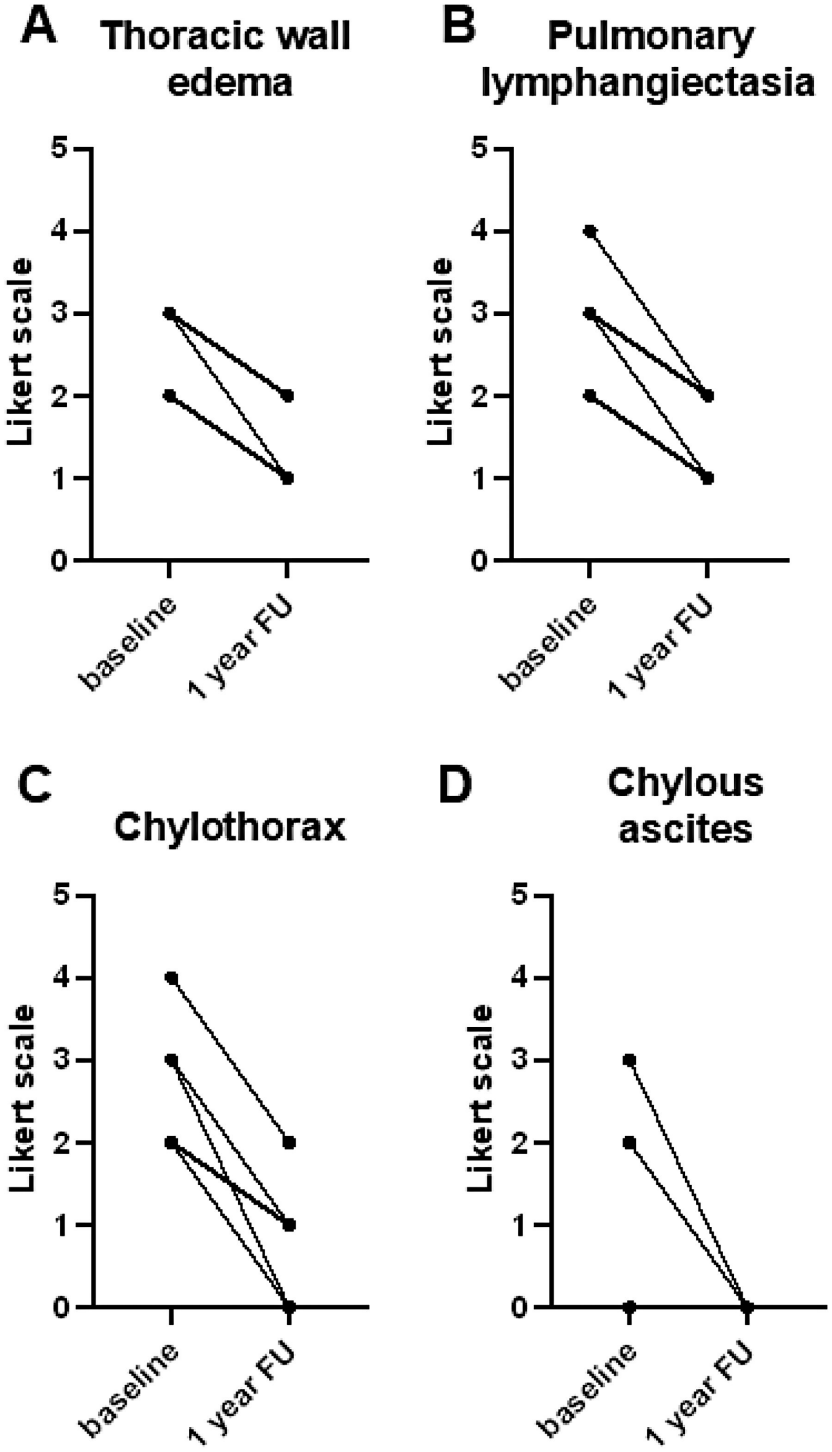
Major findings of T2-weighted MRI. Severity ratings on a 6-point-Likert scale for thoracic wall edema **(A)**, pulmonary lymphangiectasia **(B)**, chylothorax **(C)** and chylous ascites **(D)** reduced after one year of Trametinib treatment.

One year after initiation of Trametinib therapy, follow-up imaging again showed technically successful T2-weighted MRI and DCMRL in all patients (Figures 1, 2). Chylothorax completely resolved in 2/6 and was considerably reduced in the remaining 4/6 (median severity rating reduction 3→1, *p* = 0.03). Pulmonary lymphangiectasia also showed notable improvement in all patients (reduction 3→1, *p* = 0.03). Chylous ascites and chylopericardium resolved completely in the two and one affected patients, respectively. Thoracic wall edema also improved, but to a lesser degree (reduction 2→1, *p* = 0.03). See Figure 3.

Gross anatomy of the TD remained unchanged with 1/6 patients still presenting with an absent and the remaining 5/6 patients with a partially absent duct.

**Figure 4:**
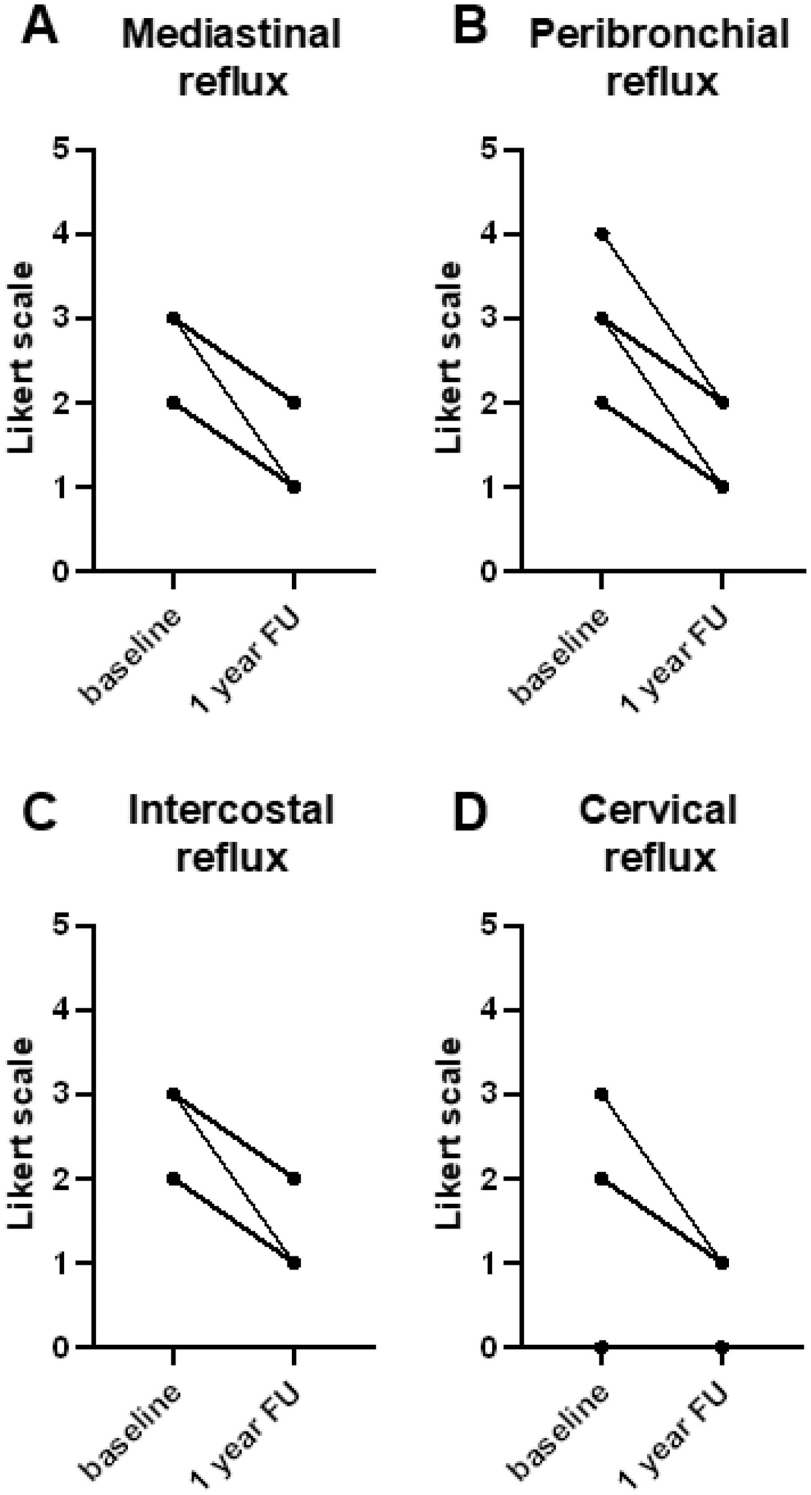
Major findings of DC-MRL. Pathological chylolymphatic reflux into mediastinal **(A)**, peribronchial **(B)**, intercostal/pleural **(C)** and cervical lymphatics **(D)** showed reduction of severity as rated on a 6-point-Likert scale throughout.

Chylo-lymphatic reflux showed considerable reduction throughout. Especially reflux into intercostal, mediastinal and peribronchial lymphatics improved substantially with a median severity rating improving from 3→1, 3→1.5 and 3→1 (each *p* = 0.03), respectively. Reflux into cervical lymphatics was still visible, but also considerably reduced in all 3/6 affected patients (Figure 4). Mild reflux into peripheral lymphatics in the groin also improved slightly.

No systematic difference in changes in imaging findings were observed between patients with pathogenic variants of *PTPN11*, *RIT1* or *KRAS*.

## Discussion

Lymphatic abnormalities are frequently observed in NS and are associated with considerable morbidity. Apart from chronic peripheral lymphedema—leading to impairment of quality of life—chylous effusions represent an acute complication that is particularly difficult to treat. Suggested treatment regimens often aim at reducing the rate of lymph fluid accumulation by medium-chain-triglyceride (MCT) diet or the application of diuretics, Propranolol, Sirolimus, Sildenafil and octreotide (6, 24). Especially for lymphatic abnormalities causing chylous effusions, interventional treatment has recently been increasingly employed (19, 25). However, considering initial experiences with poor/fatal outcomes in a number of patients with NS undergoing lymphatic embolization reported by Biko and colleagues, special care should be taken in patient-selection for interventional treatment (24). Surgical approaches such as veno-lymphatic anastomosis have been described in single case studies, but may not be applicable to all patients (26, 27). More recently, initial experiences with the MEK-inhibitor Trametinib have been described in single-case studies and small heterogeneous case series. Trametinib generally has been reported to lead to improvement or resolution of lymphatic symptoms in children and adults (1, 11, 17, 18, 28). Similar beneficial results have previously been published for NS-associated hypertrophic cardiomyopathy (13–16, 20).

The present study examined the application of Trametinib over one year in a more homogeneous single-center cohort of six infants with NS and associated severe lymphatic abnormalities including refractory chylous effusions. The main finding was that MEK-inhibition produced rapid and sustained clinical resolution or near-resolution of chylous effusions. Already within six weeks all patients showed marked clinical improvement, 5/6 patients were drain-free and all were clinically stable. After one year chylothorax was completely resolved in 2/6 and only minimal residual pleural fluid was present in 4/6 patients. This rapid clinical response mirrors the improvements described in previous case descriptions (1, 11, 28). Chylous ascites and chylopericardium also showed complete resolution, whereas peripheral lymphedema, when present, remained unchanged. However, dedicated lymphatic imaging after one year showed considerable improvement, but still markedly present lymphatic flow abnormalities. Although key functional abnormalities (i.e., reflux into intercostal, mediastinal, peribronchial and cervical lymphatic beds) diminished notably on DCMRL (median reductions typically 3→1), no patient achieved normalization of central lymphatic anatomy or flow, and enhancement of the TD remained (partially) absent in all patients at one year. Flow improvements are consistent with hypotheses that excessive ERK activation in the RAS/MAPK pathway contributes to disorganized lymphatic development, and that MEK-inhibition may facilitate reorganization (8).

This nuanced picture of imaging response to Trametinib treatment contrasts with single published case reports describing near-complete or complete imaging normalization. In this context, Dori et al. reported a teenager with NS (*SOS1*) in whom Trametinib led to resolution of PLE and chylous disease with complete and generalized remodeling of the lymphatic system within months; Li et al. described dramatic remodeling in a 12-year-old with somatic *ARAF*-mutant CCLA (1, 29). Leenders et al. reported a case of a preterm infant with *PTPN11*-pathogenic variants suffering from hydrops and refractory chylothorax with complete clinical and radiological resolution within a few weeks of Trametinib treatment. They observed reconstitution of flow through the TD hypothesizing about a restoration of lymphatic valve function (18). A further two cases (*RIT1*- and *SOS1*-pathogenic variants) described by Nakano et al. also demonstrated resolution of clinical symptoms and remodeling of the central lymphatic system (11). However, although all patients in our cohort also showed a rapid and complete resolution of clinical symptoms from chylous effusions, peripheral edema—if present—remained unchanged by Trametinib treatment over one year. More importantly, however, imaging findings in all patients in our cohort were consistently in sharp contrast to previously published results. Although there was marked improvement of central flow with less pathological reflux, these changes were far from a normalization of central lymphatic flow. This observation is particularly important in deliberations concerning the need for long-term treatment.

Several factors may account for the discrepancy between published cases and the findings in our cohort. First, disease biology may differ between different genotypes. Our cohort comprises germline *RIT1*/*PTPN11*/*KRAS*-pathogenic variants; the *ARAF*-case for example represents a somatic, mosaic, high-signal lesion where MEK-inhibition directly counters a focal driver and may permit striking macro-anatomic re-canalization. In germline NS, diffuse developmental dysplasia may be less amenable to gross structural reversal despite improved flow dynamics (29). Second, baseline anatomy demonstrates individual alterations which may lead to different treatment outcomes. Our infants started therapy all with TD absent or only segmentally formed and with extensive reflux, suggesting a system dominated by maldevelopment and maladaptive collaterals rather than discrete, sealable leaks. Functional improvements are therefore plausible without rapid macroscopic “reconstruction” of TD. The third and most likely explanation of differing results, however, remains differing methodology, image quality and therefore sensitivity of DCMRL for remaining flow abnormalities. We applied standardized high-resolution DCMRL and rigorous scoring of findings across predefined territories, which is sensitive to residual reflux even when clinically silent. Single-case narratives in contrast may reasonably emphasize gross normalization and conclusions may have been based on insufficient imaging results.

Importantly — and clinically reassuring — the observed (partial) functional remodeling of the central lymphatic system was consistent across genotypes (*RIT1*/*PTPN11*/*KRAS*) in our series, suggesting that MEK-inhibition may be effective regardless of the specific pathogenic variant. This is clinically relevant as patients with pathogenic variants in different genes can present with varied phenotypic severity, yet the underlying pathophysiologic mechanism appears responsive to targeted inhibition downstream.

These results have several implications for patient-care in infants with NS-CCLA. First, as clinical remission does not equal anatomical cure, families should be counseled that drain independence and symptom control can precede (or even outpace) structural remodeling. Residual DCMRL-abnormalities may explain why relapse can occur with dose reduction or discontinuation, and motivate cautious tapering strategies paired with imaging surveillance. Second, DCMRL proves invaluable to objectively monitor treatment effects, supporting its integration into standardized follow-up algorithms in NS lymphatic disease. Given the fact that lifetime prevalence of lymphatic abnormalities in NS-patients is around 36% (30), lymphatic evaluation using DCMRL may help to early identify those patients before they clinically manifest with e.g., chylothorax and start therapy at an early point. Third, early initiation of MEK-inhibition can be life-saving and may leverage developmental plasticity; nonetheless, the persistence of TD dysplasia/agenesis at one year tempers expectations for rapid gross anatomical reconstruction in this age group and longer follow-up intervals are needed (1, 29).

Trametinib was generally well tolerated in our cohort. Mild atopic dermatitis was the most frequently observed adverse effect. Importantly, no severe cardiac, ophthalmologic, or hematologic toxicities were observed. These findings are encouraging, particularly in the context of earlier concerns regarding Trametinibs safety profile in children (16, 20). Nonetheless, longer-term follow-up will be crucial to assess the potential risks associated with prolonged MEK-inhibition in early life.

The present study has several limitations. First, data were analyzed retrospectively in a small cohort because clinically manifest lymphatic abnormalities are rare in patients with NS. We therefore refrained from in-depth statistical analysis and the presented results should be considered as exploratory. The results of the Wilcoxon tests should be interpreted with caution given the small cohort size; nevertheless, in this exploratory analysis, reporting these results support the presence of consistent directional changes under therapy. Imaging data were analyzed by two radiologists in consensus. Further validation of the employed rating scale is warranted in future larger series. Second, the cohort was heterogeneous with respect to the underlying genetic variants and clinical manifestations. Third, only patients with symptomatic lymphatic abnormalities were included in the study. In addition, lymphatic imaging was usually performed because of refractory chylothorax. This selection process may have enriched the cohort for patients with more severe lymphatic disease and pronounced baseline imaging abnormalities, thereby limiting the generalizability of our findings to patients with milder or asymptomatic disease. The greater potential for measurable improvement in severely affected patients may also have contributed to an overestimation of the observed treatment response. Finally, although the imaging and clinical improvements were substantial, a causal effect of Trametinib cannot be established in the absence of a control group, particularly given the uncertain natural history of lymph flow abnormalities in this setting. Further, preferably multi-institution studies are needed to evaluate the incidence of—also asymptomatic—lymphatic abnormalities in patients with NS.

In summary, our findings demonstrate the potential of Trametinib to substantially alter the clinical course of infants with NS and severe lymphatic complications. Trametinib yielded rapid clinical remission of chylous effusions and measurable functional lymphatic improvement on dedicated lymphatic imaging, yet structural normalization of central lymphatic anatomy is not to be expected after one year of treatment. These exploratory findings place the favorable results of individual case reports in the broader context of diffuse developmental lymphatic dysplasia in germline NS and argue for long-term therapy and imaging-guided stewardship rather than the expectation of rapid anatomical cure. Future research should focus on prospective, multicenter studies with larger patient cohorts and longer follow-up periods to better define the safety profile and identify optimal treatment duration.

## Data Availability

The raw data supporting the conclusions of this article will be made available by the authors, without undue reservation.
